# Cognitive behaviour therapy for anxiety in adolescent with early prodromal of psychosis at boarding school

**DOI:** 10.1186/s12912-019-0360-0

**Published:** 2019-08-16

**Authors:** Niken Yuniar Sari, Budi Anna Keliat, Herni Susanti

**Affiliations:** Faculty of Nursing Universitas Indonesia, Jl Prof.Dr Bahder Djohan, Depok, Jawa Barat 16424 Indonesia

**Keywords:** Adolescent, Anxiety, Boarding- school, Cognitive behaviour therapy, Prodromal, Psychosis

## Abstract

**Background:**

Early prodromal of psychosis starts in adolescent indicated by non specific symptoms which may result in the changes of behaviour, role, function, and social interaction. Cognitive behaviour therapy is a strategic intervention in reducing anxiety symptom. The purpose of this research is to find out the effects of cognitive behaviour therapy for anxiety in adolescent with early prodromal of psychosis.

**Method:**

The design of this research was Quasi experimental design: pre-post test with control group. Samples were selected after the screening on adolescents in boarding school with the Prodromal Questionnaire (PQ16). Subjects were 78 respondents consisting of 39 each groups, obtained by random sampling technique. Nursing intervention was provided to intervention group 1, while cognitive behaviour therapy was provided to intervention group 2. The measurement of anxiety in adolescents is by using the Hamilton Anxiety Rating Scale for Anxiety (HARS). The analysis was performed by Repeated Anova and Independent t-test.

**Result:**

The result of the research shows that anxiety level decrease significantly on those two groups, whilst the decrease of anxiety and early prodromal of psychosis in the intervention group 2 indicates more decrease than intervention group 1.

**Conclusion:**

Cognitive behaviour therapy is recommended to reduce anxiety in adolescent with early prodromal of psychosis.

## Background

Early Prodromal is also known as pre onset of early psychosis phase, in which the patient experiences non-specific symptoms that may degrade perceptive, logical, and cognitive functions [1].

Adolescent suffering from early prodromal of psychosis comes at any problems that may affects his or her psychological condition [2]. Worry and anxiety are human responses when dealing with any unpleasant problems or situations that could disturb everyday life. This could lead one to suffer from early prodromal of psychosis. Anxiety is a common problem faced by teens at age of 10 to 19 years old [3]. Adolescent who suffered from anxiety are undetectable in their primary health, yet only one third of teens with anxiety were treated [4].

The most common anxiety among kids and adolescent in developing countries, was 6–20% [5]. The number of anxiety sufferers among students in Boarding School is higher (39%) than the students in Junior High School (20%) [6]. Based on their age, the result showed that the most anxiety students was at the age of 14 with prevalence of 26,9%. Students suffering from anxiety tend to disturb their daily activities, assignments, academic development, even social skill can worsen in their adult age. Accumulating problems is potential to causing depression or crisis thus affecting many aspects of life, including individual, social, or even mental aspect.

Early phase psychosis are between 15 and 25 years old [7]. Early psychosis starts in individuals whom not treated by an initial intervention [8]. It is important that Adolescent with early prodromal of psychosis suffering from anxiety be intervened with Cognitive Behaviour Therapy as soon as possible. Treating patient with this therapy can lessen his or her anxiety level or recover the symptom of psychotic [3].

Some research results prove that cognitive behaviour therapy can reduce anxiety. Severe anxiety of school-age children with thalasemia was decreased significantly after receiving the cognitive behaviour therapy [9].

The purpose of this research is to test the effect of cognitive behaviour therapy for anxiety in adolescent with early prodromal of psychosis at boarding school.

## Method

The research applies quasi experimental design: pre-post-test with control group. The subjects were 78 respondents (12 to 14 years old) in 8th grade boarding school who experienced early prodromal of psychosis and there are no drop out. Prodromal Questionnaire (PQ16) was used to screen the high risk of developing psychosis [10]. The scoring prodomal questionnaire (PQ16) the participants that endorse 6 symptom items or more are considered to be prodrmal early psychosis. Nursing intervention was applied to intervention group 1, while cognitive behaviour therapy was to intervention group 2. The measurement of anxiety in adolescents is using Hamilton Anxiety Rating Scale for Anxiety (HARS).

Techniques that are trained to respondents are to do deep breathing relaxation by breathing through the nose and out through the mouth, distraction technique by listening music, five fingers hypnosis by imagining fun things, with healthy thinking, gathering with family, achieving, being in a fun places, and spiritual techniques.

Nursing intervention cognitive behaviour therapy administered by psychiatric nurses for 5 days, nursing intervention for anxiety was delivered in both group. While each participants in control group practiced self training. The nurse continued administered cognitive behaviour therapy (CBT) in intervention group with each session lasting 30–60 min. Five days after complete CBT session, reassessment was conducted.

The analysis uses univariat and bivariat analysis with statistic test of dependent t test and Repeated Anova measure processed by computer. This research has passed ethical test by the Committee of Ethics, Nursing Faculty of Universitas Indonesia, and the researchers have passed expert validity test and competence test from the expert.

## Results

The number of adolescents suffering from early prodromal of psychosis in boarding school is 155 (48%) with 9,50% among them are on the average mean of early prodromal of psychosis. This number indicates that the adolescent in boarding school suffer mild early prodromal of psychosis.

The result finds out that the average age of adolescent is 14 years old (13.71%), the youngest is 13 years old, and the oldest is 14 years old. The proportion of male and female students are equal, which is 39 students each (50%). Most of their parents are university graduate (29.5%), with the occupational background as entrepreneur and civil servant are on the top rank, consist of 21 (26.9%). Majority of respondents are in social and economic status seen of middle-class family, based on their parents’ regular income and Regional Minimum Wage standard, which is 33 (42.3%). Moreover, 78 (100%) of respondents claim not to have genetic history, 46 (59%) claim to have ever had physical illnesses, and 44 (56.4%) claim to have had unpleasant and traumatic experiences. The most common pattern of upbringing is uninvolved, which is 57 (64.0%).

The number of adolescent suffering from early prodromal of psychosis treated by nursing for 5 days decreases from 9.50 to 7.14, as the value shown here (*p*-value < 0.001), meaning that they early prodromal of psychosis mild. Whereas, adolescents suffering from anxiety decreases from 20.26 to 13.56 as the value shown here (*p* value < 0.001), meaning that they anxiety mild The adolescents who are treated by nursing intervention shows that the mean value of early prodromal of psychosis decreases from 9.11 to 7.34, and after being independently trained, the number decreases more until 6.21 as the value shown here (*P*-value < 0.001), meaning that they mild early prodromal of psychosis. The average value of anxiety decreases from 19.00 to 13.44, and after being independently trained, the number decreases more until 9.46 as the value shown here (*P*-value< 0.001), meaning that adolescents decrease their anxiety from moderate to mild. The number of average adolescents suffering from early prodromal of psychosis decreases after being treated by nursing intervention, from 9.81 to 6.97. The number decreases more after cognitive behaviour therapy was carried out, until 5.25, as it decreases significantly (*p*-value< 0.001), meaning that the adolescent’s anxiety mild. Whereas, the average value of anxiety decreases from 21.51 to 13.69. After cognitive behaviour therapy was carried out, the number decreases more until 8.00 as the value shown here (*p*-value< 0.001), meaning that the adolescent’s anxiety decreases and becomes mild (see Table 1).

**Table 1:** Early Prodromal of Psychosis and Anxiety in Adolescents after Nursing Intervention in group 1 and after Nursing Intervention with Cognotive-Behaviour Therapy in Group 2 at Boarding School (*n* = 78)

Variable	Group	N	Mean Before Nursing Intervention	Mean After Nursing Intervention	Mean After Self Training	Mean After Nursing Intervention and CBT	Mean Diff	SD Diff	P value
Early Prodromal of psychosis	Intervention group 1	39	9.11	7.34	6.21		2.9	2.371	0.000
	Intervention group 2	39	9.81	6.97		5.25	4.56	1.693	
	Total	78	9.50	7.14			2.36	1.907	
Anxiety	Intervention group 1	39	19.00	13.44	9.46		9.54	5.419	0.000
	Intervention group 2	39	21.51	13.69		8.00	13.31	7.735	
	Total	78	20.26	13.56			6.69	5.363	

The average changing of early prodromal of psychosis and anxiety among those who are treated by nursing intervention and by cognitive-behavior therapy decreases more significantly, as the value shown here *p*-value< 0.001 than those who are treated by nursing intervention only (see Table 2).

**Table 2:** The differences of Early Prodromal of Psychosis and Anxiety in Adolescents Between Intervention Group 1 and Intervention Group 2 (*n* = 78)

Variable	Group	N	Mean	SD	SE	Mean Diff	95% CI	P value
Early Prodromal of psychosis	Intervention group 1	39	6.21	2.830	0.452	0.96	2.094–0.196	0.001
	Intervention group 2	39	5.25	2.209	0.354		2.095–0.197	
Anxiety	Intervention group 1	39	9.46	5.982	5.982	1.46	3.951–1.028	0.048
	Intervention group 2	39	8.00	5.016	5.016		3.952–1.029	

## Discussion

Screening conducted in two boarding school shows that there were 155 (48%) adolescents suffering from early prodromal of psychosis. Of 78 respondents as sampling, most of them suffer from mild early prodromal of psychosis. Early prodromal of psychosis may occur among adolescents and affect their academic performance. The occurance and the symptoms of early prodromal of psychosis starts at early adolescent and worsen at adult [11].

Early prodromal of psychosis among adolescent can be treated and recovered. Health information and education among adolescents on early prodromal of psychosis is an efficient method on how to decrease these problems.

After obtaining nursing intervention, adolescent’s anxiety had a significant decrease from 19.00 to 13.44. Nursing interventions are conducted by helping respondents to recognize their anxiety by describing the cause of anxiety, anxiety related behviour, giving opportunities to them to tell stories, searching with exploration and clarification, realizing their anxiety, and being a good listener. These interventions can help clients to solve their problems [12].

Techniques that are trained to respondents are to do deep breathing relaxation, distraction technique, five fingers hypnosis, and spiritual techniques. Breath relaxation techniques are effective in decreasing anxiety [13]. When doing relaxation breathing in the chest cavity expands so that oxygen can enter to the lungs more optimally, which then oxygen to flow throughout the body, meet the body’s oxygen demand which at the time of anxiety increases because the sympathetic nervous system is more active [14].

Distraction techniques is done by the transfer of attention to the adolescent situation that causes anxiety. Listening to music can reduce anxiety. By listening to music, one can relate to another thing that excites the most and release the tense. While the music is being played, one can distract the mind and reduce anxiety to a certain level until it is no longer existed [15]. This technique commonly known as “music therapy”, but no specific music or song is categorized as a therapy, specifically. As a distraction technique, some music can be used, and it depends on each individual’s preference [16].

The others technique is a five fingers hypnotic in decreasing anxiety. Five fingers hypnotic had an effect on the reduction of anxiety [17]. The next technique is a spiritual approach to worship. The way that some adolescents choose is to pray to God and pray at night to gain feeling of calm when facing anxiety. When a person prays, they believe they are communicating directly with God, spiritual activities can form a belief that the stressor will be able to deal with it well [18].

The result of the research shows that adolescent who are treated by nursing intervention and cognitive behaviour therapy lead to a significant changes. It should be noted that cognitive behaviour therapy conducted in this research is applied to the 8th grade students where they are in puberty. It is essential that those suffering from early prodromal of psychosis and anxiety be intervened with Cognitive Behaviour Therapy as soon as possible. Treating patients with cognitive behaviour therapy can lessen the symptoms or help the recovery [3].

The result of statistic test on the group treated by nursing shows that there is significant changes. It decreases the level of the anxiety, from moderate to mild. Interventions by nursing among adolescent can be done through assisting, identifying the anxiety, finding out its causes, and recognizing its situation.

Anxiety among adolescent shows significant changes after cognitive behaviour therapy is applied, from moderate to mild levels. This finding matches with the research on the effectiveness of Cognitive Behaviour Therapy in adolescent who are experiencing anxiety obtained that most of adolescent experience a decline in anxiety after being given Cognitive Behaviour Therapy [19].

Cognitive Behaviour Therapy is capable of handling anxiety problems, which suits with the principle of cognitive therapy believed that explains that cognitive therapy intervention is to identify, evaluate, and responds patients’ mind and false conviction thus assisting them to realize the importance of knowing the negative thoughts and to change them to be positive ones [20].

## Conclusion

This research has identified that nursing intervention can decrease the symptoms of early prodromal of psychosis and anxiety. The level of early Prodromal of psychosis among them became mild, their anxieties decreases significantly from moderate to mild.

Intervention by nursing is followed by Cognitive Behaviour Therapy thus causing the decrease of early prodromal of psychosis and anxiety’s symptoms to a significant lower level than by nursing intervention only.
